# Pre-Harvest Factors Drive Metabolic and Flavor Variations in Hainan Dayezhong Black Tea

**DOI:** 10.3390/foods15122164

**Published:** 2026-06-16

**Authors:** Zongzhuang Fang, Xiaoyan Zheng, Zhenduan Wang, Kai Guo, Xingsheng Yue, Shanying Zhang

**Affiliations:** 1 Sanya Research Institute (Hainan Laboratory Animal Research Center), Hainan Academy of Agricultural Sciences, Sanya 572019, China; 2 Institute of Tropical Bioscience and Biotechnology, Chinese Academy of Tropical Agricultural Sciences, Haikou 571101, China; 3 School of Tropical Agriculture and Forestry (School of Agricultural and Rural Affairs, School of Rural Revitalization), Hainan University, Haikou 570228, China

**Keywords:** Hainan Dayezhong black tea, geographical location, harvest season, plucking position, quality

## Abstract

This study investigated the influences of geographical origin, harvest season, and plucking position on the chemical composition and flavor characteristics of Hainan Dayezhong black tea. Systematic analysis of basic components, volatile profiles and metabolomes of tea samples collected under different pre-harvest conditions revealed significant variations in polyphenols, flavonoids and catechins, as well as distinct differences in volatile composition. Key aroma-active compounds identified were nerolidol, linalool, benzaldehyde, benzeneacetaldehyde and methyl salicylate, which were determined to be decisive for the characteristic aroma profile of Dayezhong black tea. Untargeted metabolomics further demonstrated that these factors do not merely alter individual metabolite levels, but also reprogram energy metabolism, carbon–nitrogen allocation, and secondary metabolic pathways, resulting in distinct metabolic signatures among samples. From a systematic chemical perspective, this study elucidates the metabolic basis of Hainan Dayezhong black tea quality formation and establishes a scientific foundation for targeted quality optimization through regulation of key components.

## 1. Introduction

Tea (*Camellia sinensis*) is the world’s most popular non-alcoholic beverage, consumed by over two-thirds of the global population. In China, its country of origin, tea has been cultivated and processed for more than 5000 years and is deeply integrated into national culture as the national drink [1]. Black tea, produced through fermentation, is rich in various nutrients including catechins, tea polyphenols, and amino acids. It exhibits multiple beneficial bioactive properties such as antioxidant, lipid-lowering, hepatoprotective, and anti-tumor effects, contributing positively to human health [2,3,4,5]. Despite these well-recognized health benefits, the nutritional and phytochemical profiles of tea are highly variable and are strongly influenced by numerous factors, including processing methods, harvest season, geographical location and plucking position [6,7,8].

Harvest season represents the most dynamic pre-harvest factor shaping tea quality, as it modulates the entire secondary metabolic network of tea plants through seasonal fluctuations in temperature, solar radiation, and precipitation [9]. Comparative studies have consistently shown that spring black tea accumulates higher levels of umami-enhancing amino acids and stimulatory caffeine, but lower levels of astringent catechins than summer tea, resulting in the characteristic mellow and aromatic profile of spring teas [10]. Furthermore, even within the spring season, significant compositional differences exist between early- and late-harvested teas, directly translating to variations in sensory quality [11]. Geographical origin, another dominant factor, imprints a unique chemical signature on tea through site-specific soil properties, microclimates and agronomic practices. For example, Keemun black tea possesses a distinct mineral fingerprint that allows unambiguous discrimination between different production regions [12]. Theaflavin content in black tea likewise varies with origin: samples from Yingde, Changsha and Hangzhou show a sequential decrease in theaflavin levels [13]. Furthermore, the plucking position is crucial. In Tieguanyin oolong tea, the composition and concentration of chemical compounds vary significantly with fresh leaf position, while the degree of fermentation decreases with increasing leaf maturity [14]. Nutrient elements such as nitrogen and phosphorus also show clear vertical distribution patterns within tea shoots [15]. Taken together, harvest season, origin and plucking position are key factors; they systematically affect the chemical composition, active substance content and final quality characteristics of tea.

*Camellia sinensis* var. Hainan Dayezhong is a uniquely evolved tea variety endemic to Hainan Island. Characterized by its arbor growth habit, large leaves and early sprouting phenotype, it exhibits significant genetic divergence from both *Camellia sinensis* var. sinensis and *Camellia sinensis* var. assamica, indicating rich genetic diversity [16]. As the predominant tea cultivar in Hainan Province, occupying 56.7% of the total tea cultivation area in 2023 [17], Hainan Dayezhong tea trees primarily thrive in high-altitude mountainous regions of Hainan, a tropical province located in the southernmost part of China [18]. This unique cultivation environment produces China’s sole low-latitude, high-altitude tropical black tea. Wuzhishan mountain serves as the core production area, and Wuzhishan Hainan Dayezhong black tea (WZSHDZBT) has been officially recognized as a Geographical Indication (GI) protected product.

HDZBT is rich in diverse nutritional and volatile components, including flavanols, proanthocyanidins, caffeine and flavonoid glycosides [19], whose antioxidant properties significantly contribute to the formation of its characteristic flavor [18]. Its distinctive floral and sweet notes are primarily derived from key volatile compounds, such as damascenone, benzaldehyde and linalool [20,21]. Linalool and its derivatives are the most dominant aroma constituents in HDZBT, whose contents and chiral proportions are dynamically regulated by harvest season, tissue position and processing conditions. Most notably, HDZBT exhibits a unique *S*-linalool-dominated chiral profile, which distinguishes it from all other documented Assam tea varieties [22].

However, these studies have mainly focused on processing-induced metabolic changes or single-region quality characteristics [20,21,22]. The specific regulatory effects of the three major pre-harvest factors (geographical origin, harvest season, and plucking position) on HDZBT metabolic composition and flavor characteristics have not been systematically compared. To address this critical knowledge, the effects of harvest season, geographical origin, and plucking position on key bioactive components (including total polyphenols, total catechins, total amino acids, theanine, and caffeine) and volatile compounds in HDZBT were investigated through comparative analysis of representative samples. By elucidating the compound accumulation patterns and flavor variations driven by these factors, and defining the resultant chemical compositional differences, this study provides essential empirical evidence to support geographical traceability and quality improvement of HDZBT.

## 2. Materials and Methods

### 2.1. Materials

The picking locations, seasons and parts of the five Hainan Dayezhong fresh samples are summarized in Table 1. The fresh leaves were withered, rolled, fermented and dried to make Dayezhong black tea. The specific processing steps were carried out according to the method of Hu et al. (2024) [18]. All samples were stored at −20 °C in sealed containers for further experiments.

### 2.2. Reagents and Chemicals

Standard compounds, including (+)-catechin (+C, 99.8%), epigallocatechin (EGC, ≥98%), epicatechin (EC, ≥98%), epicatechin gallate (ECG, ≥98%), and epigallocatechin gallate (EGCG, ≥98%), were purchased from Shanghai Yuanye Bio-Technology Co., Ltd., Shanghai, China. Caffeine (≥98%), ethyl decanoate (≥98%), l-aspartic acid (Asp, ≥98%), l-glutamic acid (Glu, ≥99%), l-proline (Pro, ≥99%), l-serine (Ser, ≥99%), l-alanine (Ala, ≥98%), l-glycine (Gly, ≥99%), l-threonine (Thr, ≥98%), l-arginine (Arg, ≥98%), l-phenylalanine (Phe, ≥98%), l-leucine (Leu, ≥98%), l-valine (Val, ≥98%), l-histidine (His, ≥99%), l-lysine (Lys, ≥98%), l-methionine (Met, ≥98%) and l-tyrosine (Tyr, ≥99%) were obtained from Shanghai Sigma-Aldrich Trading Co., Ltd., Shanghai, China. All other reagents were verified to be of analytical or chromatographic grade.

### 2.3. Sensory Evaluation

Sensory evaluation was carried out in accordance with Cao et al. (2019) with some modifications [23]. The method was to weigh 2 g of tea sample, add 100 mL of boiling water, and brew for 5 min. The brewed infusions were immediately presented to a trained panel consisting of 10 tasters (5 males and 5 females). Each panelist evaluated the samples independently in individual booths to eliminate mutual interference, and the entire sensory analysis was replicated three times on separate days. A weighted scoring system with a maximum score of 100 was used, with scores allocated to five attributes: appearance (25%), soup color (10%), aroma (25%), taste (30%) and infused leaves (10%). The taste attribute was further subdivided into five independent sub-attributes with the following weight distribution: sweetness (6%), bitterness (6%), astringency (6%), umami (6%) and aftertaste (6%). Each sub-attribute was scored on a 0–100 scale by the panelists, and the comprehensive taste score was calculated as the weighted average of these five sub-attributes. The radar diagrams were drawn using the final calculated scores of the five primary attributes.

### 2.4. Physicochemical Analysis

The ash content was determined according to China National Standard GB 5009.4-2016 [24]. Soluble protein content was determined via the Coomassie Brilliant Blue G-250 assay [25], with absorbance read at 595 nm (Molecular Devices, SpectraMax iD3, San Jose, CA, USA) and bovine serum albumin as the reference standard. The soluble sugar content was determined by the phenol–sulfuric acid colorimetric method [26]: cryogenically ground tea samples were extracted, and the extracts were reacted with 9% phenol and concentrated sulfuric acid. Absorbance was read at 485 nm using a SpectraMax iD3 (Molecular Devices) microplate reader, with glucose as the calibration standard. Flavonoid content was determined according to the spectrophotometric method [27], and the total phenol, catechin and caffeine contents were measured according to China National Standard GB/T 8313-2018 [28]. Briefly, tissue (1.0 g) was homogenized on ice with 5 mL of 80% ethanol and centrifuged at 8000× *g* for 10 min at 4 °C. Total phenol content was determined by the Folin–Ciocalteu method at 765 nm with gallic acid as standard, while flavonoid content was measured by the aluminum nitrate colorimetric method at 510 nm using rutin for calibration. Results are expressed as mg gallic acid equivalents g^−1^ FW and mg rutin g^−1^ FW, respectively.

A 2 mL supernatant was further diluted to 10 mL with EDTA-2Na/ascorbic acid/acetonitrile stabilizing solution and filtered through a 0.22 μm nylon filter. Catechin contents were determined by HPLC (LC-20 A, Shimadzu, Kyoto, Japan) equipped with a C_18_ column (ECOSIL, 5 μm, 250 × 4.6 mm) at 35 °C and a flow rate of 1.0 mL/min. Mobile phase A (90 mL acetonitrile, 20 mL acetic acid, 2 mL 10 mg/mL EDTA-2Na, water to 1000 mL), B (800 mL acetonitrile, 20 mL acetic acid, 2 mL 10 mg/mL EDTA-2Na, water to 1000 mL): 100% A for 10 min, linear decrease to 68% A in 15 min, hold for 10 min, then return to 100% A. Detection at 278 nm. Quantification by external standard curves of six catechins (GA, +C, EGC, EC, EGCG, ECG).

### 2.5. Amino Acid Analysis

The determination of amino acids in HDZBT was performed according to Xue et al. (2023) with modifications [29]. Briefly, tea samples of 0.2 g were ground by a freezer grinder and weighed, and extracted with 1 mL deionized water ultrasonication for 30 min, followed by centrifugation (Z218MK, Hermle, Gosheim, Germany) at 12,000× *g* for 10 min. The supernatant was taken and passed through a 0.22 μm polytetrafluoroethylene filter membrane. Analysis was performed on an UPLC-MS-MS (TripleTOF 6600, AB Sciex, Concord, ON, Canada) equipped with a C18 column (3.9 mm × 150 mm, 4 μm). The mobile phase consisted of water (A) and methanol (B), and the gradient elution program was as follows (*v*/*v*): 0–0.5 min, 99% A; 0.5–2.5 min, 92% A; 2.5–15 min, 50% A; 15–18 min, 5% A; 18–20 min, 99% A. Quantification was performed using a mixed standard solution of 18 amino acids.

### 2.6. Determination of Volatile Compounds in Dayezhong Black Tea

Volatile extraction from each sample was performed using fully automated headspace solid-phase microextraction (HS-SPME). Volatile compound analysis was conducted according to Wang et al. (2023) with minor modifications [30]. Specifically, cryogenically ground tea sample (0.4 g) was placed in a 20 mL headspace vial, infused with 2 mL boiling water, and supplemented with 1200 μL of 0.9% sodium chloride solution and 10 μL of ethyl decanoate standard solution (0.02 mg/mL). The mixture was equilibrated at 60 °C for 5 min with agitation, followed by 30 min HS-SPME extraction using a pre-conditioned fiber (50/30 μm DVB/CAR/PDMS).

The SPME fiber was introduced into an Agilent GC-MS (7890A-5975C) system (Santa Clara, CA, USA ) equipped with an HP-5 column (30 m × 0.32 mm × 0.25 μm). Chromatographic conditions were programmed as follows: initial oven temperature: 50 °C (hold 3 min); ramp 1: 10 °C/min to 150 °C (hold 3 min); ramp 2: 10 °C/min to 250 °C (hold 10 min). Mass spectrometric detection was operated with electron impact (EI) ionization under these parameters: ion source temperature: 230 °C, quadrupole temperature: 220 °C, transfer line temperature: 280 °C, electron energy: 70 eV, scan range: *m*/*z* 35–500.

Volatile compounds were identified by two complementary independent methods: mass spectral matching against the NIST 11 library with a minimum matching threshold of 80%, and retention index (RI) calibration using a C7–C30 n-alkane mixture analyzed under identical chromatographic conditions, with only compounds showing RI deviations within ±20 relative to literature values retained for further analysis. All reported volatile compounds were identified at Metabolomics Standards Initiative (MSI) Level 2 confidence, and their relative concentrations were quantified by normalization to the ethyl decanoate internal standard. Relative odor activity values (ROAVs) were calculated according to Chen et al. (2024) [31].

### 2.7. Non-Targeted Metabolomics

Quantitative analysis of broad-target metabolites was performed by Genedenovo Biotechnology Co., Ltd. (Guangzhou, China). Tea samples were lyophilized, followed by cryogenic grinding (MM 400, Retsch; 30 Hz, 1.5 min). Subsequently, 100 mg of tea powder was weighed and extracted with 500 μL of 80% aqueous methanol through vortex mixing. The extract was centrifuged at 15,000× *g* for 20 min, the supernatant was filtered through a 0.22 μm nylon filter membrane, and the subsequent extract was analyzed by UPLC- MS/MS (QTRAP 6500+, SCIEX, Concord, ON, Canada) equipped with a column (2.5 μm, 2.1 mm × 150 mm, Xselect HSS T3, Waters).

The UPLC–HR-MS/MS analysis was performed under the following conditions: mobile phase: A-0.04% acetic acid in water, B-0.04% acetic acid in acetonitrile; gradient program A:B (*v/v*): 0 min: 95%/5%, 11.0 min: 5%/95%, 12.0 min: 5%/95%, 12.1 min: 95/5%, 15.0 min: 95%/5%; flow rate: 0.4 mL/min; column temperature: 40°C; injection volume: 2 μL. Mass spectrometric parameters were optimized based on the ESI-QTRAP-MS method described by Chen et al. (2013) [32]. To ensure the reliability of metabolomics data, a pooled quality control (QC) sample was prepared by mixing equal volumes of all sample extracts. Relative standard deviation of peak areas for all features in QC samples < 30%. The bioinformatics data of metabolite determination results were obtained by the Omicsmart platform (https://www.omicsmart.com). The detailed non-targeted metabolomics analysis method is shown in the Supplementary Materials.

### 2.8. Data Analysis

Statistical analysis was performed using SPSS Statistics 25.0 (IBM, Armonk, NY, USA). One-way analysis of variance (ANOVA) was conducted, followed by Duncan’s multiple range test for significance determination. Significantly differential metabolites were screened based on the following criteria: |log_2_ (fold change)| > 1, variable importance in projection (VIP) score > 1.0, *p*-value < 0.05. Data visualization was implemented using Origin 2023 (OriginLab Corp., Northampton, MA, USA). All experiments were replicated three times, with results expressed as mean ± standard deviation. Statistical significance was defined at *p* < 0.05.

## 3. Results and Discussion

### 3.1. Sensory Evaluation

**Figure 1:**
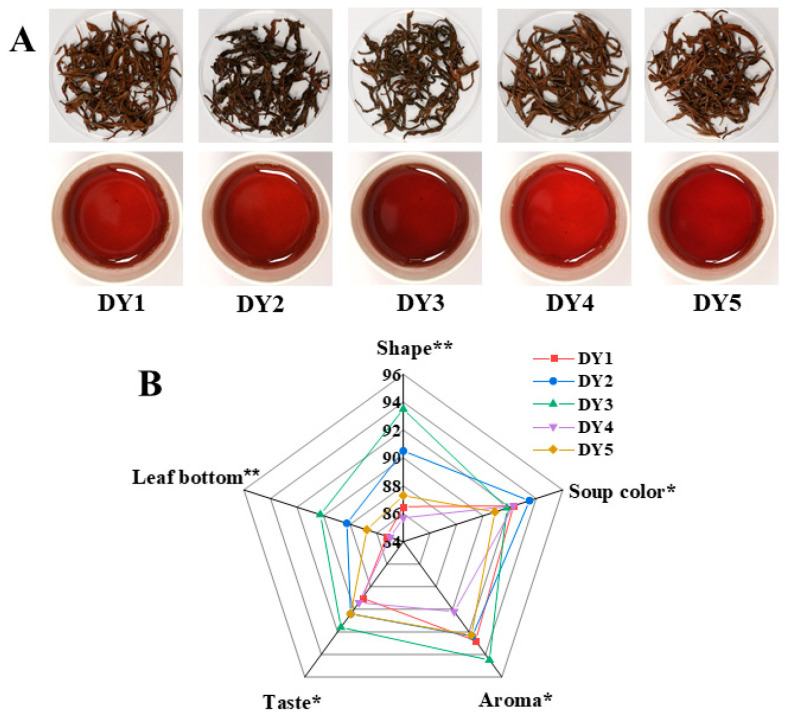
Appearance (**A**) and radar map of sensory attributes (**B**) of 5 tea samples. Statistical significance was determined by one-way ANOVA followed by Duncan’s post hoc test: *p* < 0.05 (*), *p* < 0.01 (**).

The appearance serves as a critical indicator of tea quality. All five HDZBT samples exhibited a characteristic golden ring upon infusion (Figure 1A). The sensory evaluation radar chart (Figure 1B) comprehensively presents the five primary sensory attributes evaluated in this study, appearance, soup color, aroma, taste, and infused leaves, with the comprehensive taste score (accounting for 30% of the total sensory score) calculated as the weighted average of five independent taste sub-attributes: sweetness, bitterness, astringency, umami and aftertaste. The comprehensive quality scores were ranked in descending order as follows: DY3 > DY2 > DY5 > DY1 > DY4. Notably, the spring-harvested sample DY3 demonstrated superior sensory quality compared to summer-harvested counterparts (*p* < 0.05), which was characterized by tightly rolled and uniform infused leaves, a bright indigo-red liquor color, robust taste and intense aroma. Marked differences in liquor color were observed, which correlated with variations in theaflavin and thearubigin contents: DY3 had the darkest ruby-red liquor, while DY4 showed the lightest orange-red color. Additionally, significant geographical variations were identified, with teas from the WZS region scoring significantly higher in both aroma and taste attributes compared to samples from other regions (*p* < 0.05). This quality difference may be associated with the unique microclimate characteristics of the WZS region, which is a core high-altitude tea-producing area in Hainan Island with an average cultivation altitude of 600–800 m, mild spring temperatures (18–25 °C), abundant diffuse sunlight, and high relative humidity [18,33]. These environmental conditions have been widely reported to promote the accumulation of flavor-related metabolites in tea plants, consistent with our finding that DY3 contained higher levels of soluble sugars, total flavonoids and ascorbic acid.

### 3.2. The Chemical Components of Dayezhong Black Tea

Primary chemical components in tea, such as soluble sugars, total flavonoids, total polyphenols, and free amino acids, are core determinants of flavor and quality of black tea [34]. The biosynthesis and accumulation of these primary and secondary metabolites are strongly modulated by harvest season, plucking position and geographical location [35]. Accordingly, the present study quantified seven fundamental chemical indicators, namely ash, total flavonoids, soluble sugars, ascorbic acid, total polyphenols, soluble proteins and free amino acids, across HDZBT samples with distinct harvest seasons, geographical origins and plucking position.

**Figure 2:**
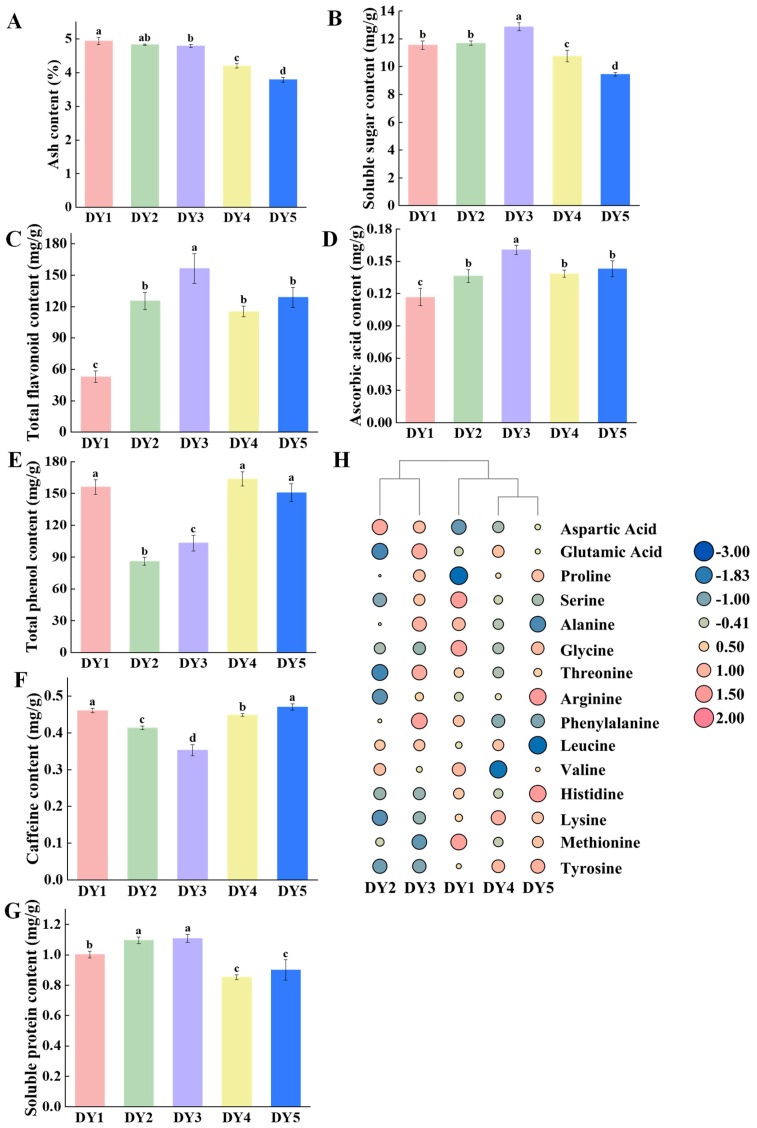
Contents of chemical components in Hainan Dayezhong black tea. (**A**) Ash content, (**B**) soluble sugar content, (**C**) total flavonoid content, (**D**) ascorbic acid content, (**E**) total polyphenol content, (**F**) caffeine content, (**G**) soluble protein content, (**H**) free amino acid content. Different letters indicate significant differences among treatments (*p* < 0.05).

Ash content differed markedly among all samples, following the order DY1 > DY2 > DY3 > DY4 > DY5 (Figure 2A). Regional and seasonal comparisons showed that WZS teas had lower ash levels than QZ teas. Spring-harvested samples contained less ash than summer-harvested counterparts. For samples from the same WZS region but with different plucking standards, the one-bud-and-two-leaf sample (DY4) possessed significantly higher ash content than the tender bud sample (DY5). Tea ash content is well recognized to be negatively correlated with fresh leaf tenderness [36]. These results clearly indicate that: (1) fresh tea leaves from WZS display better tenderness, and (2) for tea from the same producing area (QZ), tender buds contribute to much higher tenderness of finished tea compared with mature one-bud-and-two-leaf shoots. In conclusion, tender buds collected from the WZS production area are optimal raw materials, which endow HDZBT with superior tenderness and comprehensive quality.

The comprehensive taste score shown in the radar chart (Figure 1B) represents the weighted average of five independent taste sub-attributes: sweetness, bitterness, astringency, umami and aftertaste. Significant variations in soluble sugar content were observed among Dayezhong black tea samples, with DY3 exhibiting the highest level and DY5 the lowest (Figure 2B). Soluble sugars are primary contributors to the sweet taste of tea infusions. Total flavonoids, ascorbic acid and total polyphenols are essential functional components that modulate black tea flavor and confer antioxidant functionality. As a crucial natural antioxidant, ascorbic acid participates in a series of redox reactions during tea processing, thereby affecting the final flavor formation of black tea [37]. Notably, DY3 showed considerably higher contents of total flavonoids and ascorbic acid than the other tested samples, while its total polyphenol content was comparable to DY2 and lower than DY1, DY4 and DY5 (Figure 2C–E). This indicates that spring-harvested Dayezhong black tea from the WZS region exhibited a distinct antioxidant metabolite profile rather than a general accumulation of all antioxidant compounds. In addition, higher levels of total flavonoids and ascorbic acid were recorded in the spring sample (DY3) relative to the summer-harvested counterpart (DY2). Such metabolic discrepancies may be partially associated with mild low-temperature stress in spring environments, which potentially activates the phenylpropanoid pathway and facilitates the biosynthesis and accumulation of flavonoids and polyphenol derivatives [38]. This metabolic distinction may further confer relatively strong antioxidant properties to spring Dayezhong black tea. Caffeine, an important alkaloid in tea, is closely associated with bitter taste perception and also exerts multiple physiological functions such as anti-fatigue and metabolic regulation [30]. In this study, DY1 and DY3 contained the highest levels of caffeine, followed by DY2, DY4 and DY5 (Figure 2F). As a major contributor to black tea bitterness, caffeine levels correlated well with sensory scores. DY1 and DY3 had significantly higher taste scores (*p* < 0.05) than the other samples. This suggests that differences in caffeine content partially explain the variations in bitterness intensity among samples. Collectively, these metabolites shape DY3’s characteristic taste: initial bitterness with a pleasant lingering sweet aftertaste.

Although soluble proteins are relatively low in fresh tea leaves, they play a vital role in enhancing the fullness and viscosity of tea infusions. They also act as critical precursors for free amino acid synthesis, especially those contributing to fresh and sweet flavors [38]. Soluble protein contents varied across the five HDZBT samples (DY1–DY5). DY3 and DY2 presented higher soluble protein levels, followed by DY1, whereas DY4 and DY5 accumulated relatively lower contents (Figure 2G). Soluble protein accumulation was closely related to geographical conditions. This represented a key environmental driver of metabolic differences in this tea cultivar. Changes in soluble protein abundance may affect amino acid metabolism, thus potentially modulating the overall flavor of tea products [39]. Free amino acids are indispensable for shaping the delicate flavor nuances of black tea. Accordingly, the contents of umami, sweet, and bitter amino acids were quantitatively determined to clarify flavor-related metabolic differences among samples (Figure 2H). DY3 possessed the highest concentrations of umami amino acids (Asp and Glu, 46.24 mg/g) and sweet amino acids (Pro, Ser, Ala, Gly and Thr, 165.28 mg/g). By comparison, DY2 had the greatest accumulation of bitter amino acids (Arg, Phe, Leu and Val, 618.90 mg/g). Although bitter amino acids in DY3 also reached a relatively high level (600.91 mg/g), the abundant umami and sweet amino acids in this sample could offset bitter sensations, forming a milder and more harmonious taste perception. Furthermore, during black tea fermentation and drying, free amino acids undergo Maillard reactions with reducing sugars, and the reactions produce caramel and sweet aromatic compounds [29]. They can also mitigate bitterness and astringency induced by tea polyphenols, caffeine and bitter amino acids, thereby facilitating the formation of a mellow and balanced sensory profile of black tea.

### 3.3. Effects of Various Factors on Catechin Content in Dayezhong Black Tea

**Figure 3:**
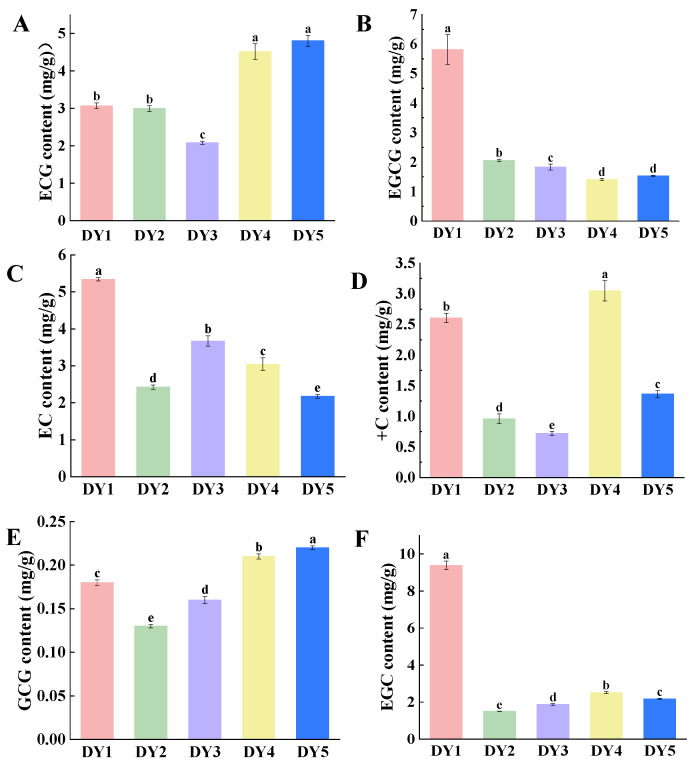
Catechin contents in Hainan Dayezhong black tea. (**A**) EGC content, (**B**) EGCG content, (**C**) EC content, (**D**) (+)-C content, (**E**) GCG content, (**F**) ECG content. Different letters indicate significant differences among treatments (*p* < 0.05).

Catechins are the primary polyphenolic compounds in tea, and serve as key secondary metabolites responsible for the astringency and bitterness of tea infusions [40]. To investigate the effects of geographical origin, harvesting season and plucking position on the catechin composition of HDZBT, six major catechin monomers—EGC, EGCG, EC, (+)-C, GCG and ECG—were analyzed (Figure 3). Individual catechin monomers exhibited distinct accumulation patterns among treatments. EGC was lowest in DY3 and highest in DY4 and DY5 (Figure 3A). As the core esterified catechin, EGCG displayed the trend of DY1 > DY2 > DY3 > DY4, with the spring sample from BS (DY1) presenting the maximum concentration. For teas from WZS, higher EGCG was detected in the summer sample (DY2) relative to the spring counterpart (DY3), whereas plucking position exerted no obvious influence on EGCG accumulation (Figure 3B). EC content decreased in the order DY1 > DY3 > DY4 > DY2 > DY5 (Figure 3C), while (+)-C content followed the order DY4 > DY2 > DY5 > DY1 > DY3 (Figure 3D). GCG was most abundant in DY5, followed by DY4, DY1, DY3 and DY2 (Figure 3E), and ECG was highest in DY1, with a descending sequence of DY4 > DY5 > DY3 > DY2 (Figure 3F). Overall geographical conditions appeared to be the dominant factor associated with differential catechin accumulation, and samples from BS (DY1) possessed higher abundances of key esterified catechins such as EGCG. Seasonal effects varied greatly depending on compound type and cultivation region, as illustrated by the higher summer EGCG level within the WZS production area. Plucking position mainly altered the accumulation of certain individual monomers such as GCG, with negligible effects on EGCG content.

Catechins represent major end products of the flavonoid/anthocyanin sub-branches in the phenylpropanoid pathway. During black tea fermentation, catechins are susceptible to oxidation and polymerization to generate typical oxidized polyphenols, including theaflavins, thereby modulating tea liquor color and sensory flavor characteristics [29,35]. Differential accumulation of esterified catechins across tea germplasms is closely linked to the activity of C-ring acyltransferases (SCPL-ATs), which function as key catalysts for the biosynthesis of ester-type catechins such as EGCG [41]. Furthermore, environmental factors and processing temperature can reshape catechin profiles: they regulate the activities of pathway-related synthases and the epimerization efficiency of catechin monomers. Previous studies have indicated that summer and autumn teas generally contain higher total catechin levels than spring teas, largely driven by distinct light and temperature regimes [35]. The present results are consistent with such trends, as the summer-harvested WZS sample (DY2) showed higher EGCG content than its spring counterpart (DY3). This seasonal difference implied that high temperature and intense solar radiation may potentially accelerate phenylpropanoid metabolism and alter the activity of key synthetases such as SCPL-AT, thereby facilitating EGCG biosynthesis. Notably, this deduction remains speculative here, and further enzymatic and molecular biological evidence is still required for confirmation.

Combined with the above geographical variation, the present data further suggest that geographical origin establishes the inherent accumulation potential of EGCG, as reflected by the high EGCG level in the spring sample from BS (DY1). Within the same producing region (e.g., WZS), seasonal fluctuation acts as a critical regulator of catechin metabolism, with summer conditions appearing more favorable for EGCG enrichment. By contrast, plucking position exerted no obvious influence on EGCG abundance, and may primarily mediate the accumulation of other catechin monomers such as GCG. Collectively, geographical origin, harvest season and plucking position jointly shape the catechin profiles of HDZBT: geographical background determines intrinsic metabolic potential, seasonal conditions modulate biosynthetic intensity, and plucking position regulates the relative distribution of individual catechin compounds [33]. It should be pointed out that theaflavins and thearubigins are formed through the oxidation and polymerization of catechins during the fermentation process of black tea, and play a crucial role in forming the final flavor characteristics. These oxidized polyphenols have a significant impact on the astringency, bitterness and color of black tea brewing. This study mainly focuses on the influence of major non-oxidative metabolites on flavor characteristics. The quantitative determination of theaflavins and thearubigins and their specific contributions to HDZBT flavor will be systematically studied in our future research.

### 3.4. Correlation Analysis Between Chemical Components and Sensory

**Figure 4:**
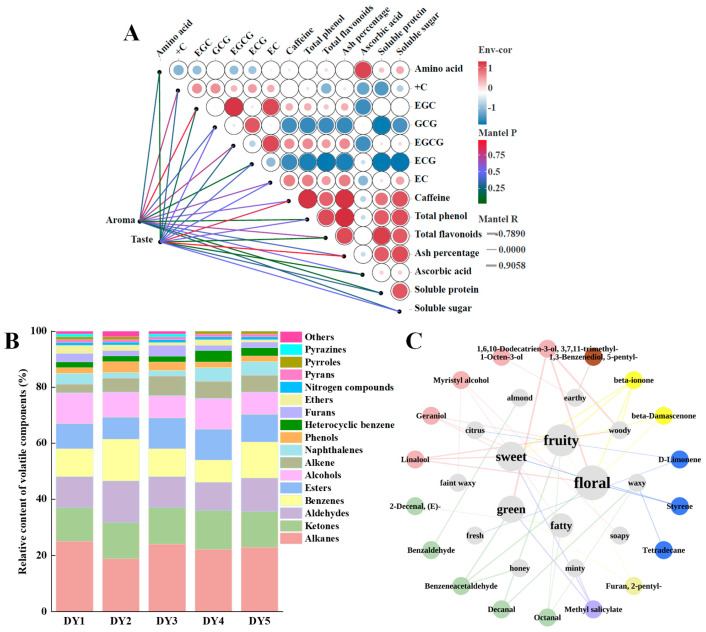
Correlation analysis between characteristic components and chemical properties of Hainan Dayezhong black tea (**A**), classification of volatile compounds (**B**), and association network between sensory attributes and differential aroma-active compounds (**C**). Gray nodes represent sensory attributes, outer nodes represent aroma-active compounds; line width is proportional to ROAV. Node size corresponds to the degree of the sensory attribute. Line color corresponds to the node color of the aroma compound.

The Mantel test revealed the overall correlation between chemical components and sensory attributes [42]. The detailed correlation network among components and their specific links to sensory dimensions were also examined. Together, these results systematically clarify the multi-level chemical basis of flavor formation in HDZBT (Figure 4A). This indicates that the flavor profile of black tea arises from synergistic interaction of all chemical components rather than the contribution of any single compound. Further analysis revealed that aroma attributes were strongly positively correlated with soluble protein and total phenol contents, suggesting a potential key role of these components in the formation, stabilization or coordinated release of volatile aromatic compounds. In contrast, certain catechins such as ECG and GCG showed negative correlations with aroma, reflecting their potential inhibitory effects on specific aroma precursors or volatile pathways during processing [43]. Taste attributes were significantly positively correlated with amino acids, ascorbic acid and total flavonoids, which collectively form the material basis for the freshness, fullness and complex aftertaste of tea infusions. The consistent significance of ascorbic acid and total flavonoids further underscores their stable contribution to taste perception. Moreover, the inter-component correlation matrix revealed an intrinsic synergistic–antagonistic regulatory network. Caffeine exhibited strong positive correlations with total phenols, total flavonoids and soluble sugar, suggesting possible co-accumulation pathways during metabolism or processing [38]. In contrast, ECG showed strong negative correlations with total flavonoids, soluble protein and soluble sugar, indicating potential competitive or substitutive relationships with these flavor-positive components during biochemical transformation. Catechin monomers also displayed clear divergence: EGC, EGCG and EC were highly positively correlated with each other and shared accumulation trends with caffeine and total phenols. By contrast, GCG and ECG were generally negatively correlated with these components. These differences further modulate the intensity and quality of bitterness and astringency, thus shaping overall flavor balance [38]. In summary, the characteristic flavor of HDZBT is shaped by two aspects. One is the macro-scale association between overall chemical composition and sensory attributes. The other is the specific contributions of key components within a complex synergistic–antagonistic. network.

### 3.5. Analysis of Volatile Components

Volatile components are the fundamental material basis determining tea aroma, and differences in their types and concentrations directly shape distinct aroma type characteristics. In this study, a total of 215 volatile compounds were identified in HDZBT samples. Based on their chemical structures, these compounds were classified into categories including alkanes, ketones, aldehydes, benzenes, esters, alcohols, alkenes, naphthalenes, phenols, heterocyclic benzenes, furans, ethers, nitrogen-containing compounds, pyrans, pyrroles and pyrazines, which collectively accounted for over 98% of the total VOCs (Figure 4B). Significant differences were observed in both the variety and relative concentrations among samples. DY3 contained the richest variety (128), followed by DY2 (122), DY4 (116), DY5 (116) and DY1 (96). The 15 most abundant VOCs constituted the core aromatic profile of HDZBT, including linalool, methyl salicylate and benzeneacetaldehyde.

Hierarchical cluster analysis based on VOCs revealed that the five HDZBT samples were clearly divided into two major distinct clusters (Figure S1), rather than grouping strictly according to a single factor of geographical origin, harvest season or plucking position. This indicates that the overall volatile profile of HDZBT is shaped by the intertwined effects of multiple pre-harvest factors. Cluster I comprised DY1, DY2 and DY3 samples, among which DY2 and DY3 first formed a sub-cluster, indicating that their volatile metabolic profiles were the most similar among all samples. Cluster II consisted of DY4 and DY5 samples, which showed highly consistent volatile composition characteristics. Within Cluster I, the preferential aggregation of DY2 and DY3 (both from the WZS) region, despite different harvest seasons) indicated that geographical origin exerted a stronger influence on the overall volatile profile than seasonal variation within the same plucking position. The subsequent merging of DY1 (from the BS region, spring-harvested one-bud-and-two-leaf) into Cluster I further confirmed that plucking position was the dominant factor determining the global volatile metabolic signature, overriding the effect of geographical origin to some extent. The close clustering of DY2 and DY3 may be attributed to their shared WZS-origin characteristic volatile components, such as specific alkanes and esters. The similarity between DY4 and DY5 could be explained by their common geographical origin (QZ) and harvest season (summer), which led to a similar background metabolite spectrum despite differences in plucking position. The aggregation of DY1 with the WZS one-bud-and-two-leaf samples may be associated with comparable proportions of certain medium-abundance aroma components (e.g., alkenes and aldehydes) among these three samples. Collectively, these results demonstrate that the volatile profile of HDZBT is co-determined by the synergistic effects of geographical origin, harvest season and plucking position. Among these factors, plucking position exerts the most pronounced influence on the global volatile metabolic pattern, followed by geographical origin, while the effect of harvest season is relatively weaker and mainly manifested within specific geographical and plucking position contexts. The final aroma profile is co-determined by the synergistic effect of “origin-characteristic components” and “cross-origin shared components”.

Relative odor activity value (ROAV) analysis is an effective method for screening key VOCs in tea. VOCs with ROAV > 1 are conventionally regarded as major contributors to overall aroma [44]. In this study, 18 aroma-active VOCs with ROAV > 1 were screened from 46 key differential VOC, including tetradecane, 1,3-benzenediol, 5-pentyl-, nerolidol (1,6,10-dodecatrien-3-ol, 3,7,11-trimethyl), 1-octen-3-ol, myristyl alcohol, geraniol, linalool, (E)-2-decenal, benzaldehyde, benzeneacetaldehyde, decanal, octanal, β-damascenone, β-ionone, methyl salicylate, styrene, 2-pentylfuran and D-limonene (Table S1). These substances collectively contribute to the characteristic floral, fruity, woody and nutty notes of HDZBT. Regarding the contribution to aroma attributes (Figure 4C), the number of active compounds associated with a fruity scent was the highest (8), followed by floral (7) and sweet (7) notes. Subsequently, these were followed by waxy (4), green (3), woody (3), fatty (3), and earthy (3) notes, while nutty and herbal notes were each associated with one compound. Nerolidol, which exhibited the highest ROAV in this study, was positively correlated with both fruity and floral attributes, followed by linalool. Both were identified as core aroma-active compounds shaping the characteristic profile of the samples [45]. Benzaldehyde, benzeneacetaldehyde and methyl salicylate also exhibited relatively high ROAVs, contributing to the cherry-like, rose-like and sweet orange-like aromas of HDZBT [46]. Most notably, styrene was exclusively detected in WZS samples (DY2: 9.23, DY3: 2.95) and completely absent in samples from other regions, making it a unique diagnostic marker for WZSBT. The key aroma VOCs identified in this study showed certain differences from those reported in three other WZSBTs, which may be attributed to variations in harvesting season and tea cultivar [21].

### 3.6. Metabolomic Analysis of Dayezhong Black Tea

**Figure 5:**
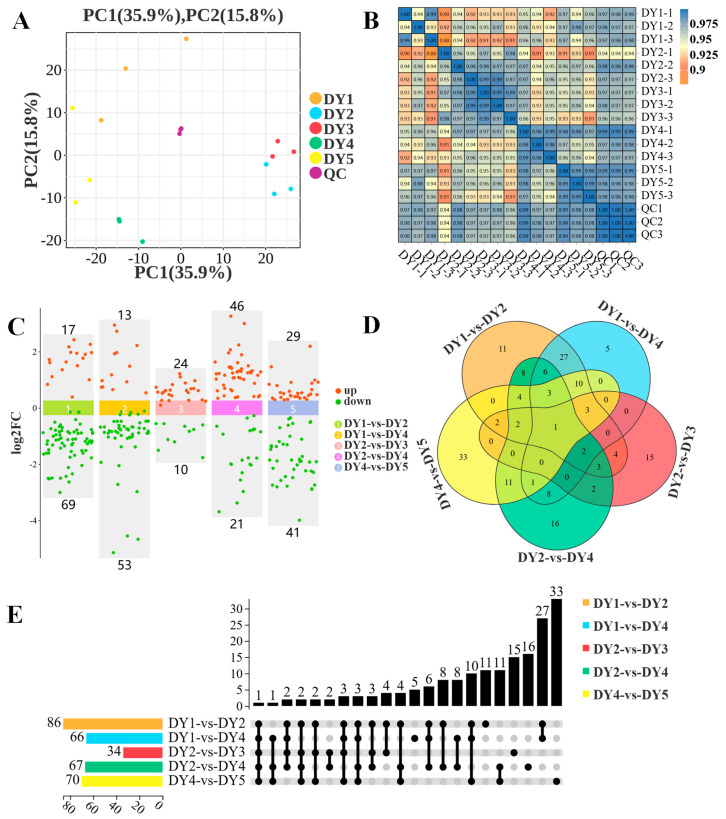
Metabolome analysis of Hainan Dayezhong black tea. (**A**) Principal component analysis (PCA) score plot, (**B**) Pearson correlation analysis plot among samples, (**C**) distribution of up- and downregulated differential metabolites in the five comparison groups, (**D**) Venn diagram of differential metabolites, (**E**) intersection analysis of metabolites across different comparison groups.

An untargeted metabolomics approach was employed to investigate the integrated effects of harvest season, geographical origin and plucking position on the metabolic profiles of HDZBT. A total of 177 differential metabolites were identified across all comparison groups (Table S2), primarily including flavonoids, phenolic acids, lipids, amino acids and derivatives, organic acids and carbohydrates. These compositional findings are consistent with previously reported metabolite profiles of HDZBT [29]. Principal component analysis (PCA) revealed that the first two principal components accounted for 51.7% of the total variance, clearly separating the DY1, DY2, DY3, DY4 and DY5 groups (Figure 5A). Pearson correlation coefficients between samples were all above 0.91 (Figure 5B), indicating high experimental reproducibility. A bar chart illustrates the distribution of upregulated and downregulated differential metabolites across comparison groups (Figure 5C). Specifically, the numbers of upregulated metabolites in the DY1-vs-DY2, DY1-vs-DY4, DY2-vs-DY3, DY2-vs-DY4 and DT4-vs-DY5 groups were 17, 13, 24, 46, and 29, respectively, while the corresponding downregulated metabolites numbered 69, 53, 10, 21 and 41. Venn diagram analysis showed that norvaline was the only differential metabolite shared across all comparison groups (Figure 5D). Further overlap analysis of differential metabolites via an upset plot revealed several shared metabolites across different comparison groups (Figure 5E), suggesting common metabolic responses among these groups.

KEGG pathway enrichment analysis revealed that 12, 13, 11, 8 and 10 metabolic pathways were significantly enriched in the DY1-vs-DY2, DY2-vs-DY3, DY1-vs-DY4, DY2-vs-DY4, and DY4-vs-DY5 comparison groups (Figure S2). These differentially enriched pathways systematically underscore the profound impact of harvest season, plucking position and geographical origin on the HDZBT metabolic profiles. Significantly enriched pathways were primarily involved in several core biological processes, including primary metabolism, secondary metabolism, and signal transduction and stress response. Key pathways included amino acid and nitrogen metabolism (e.g., alanine, aspartate and glutamate metabolism; lysine degradation; aminoacyl-tRNA biosynthesis), lipid metabolism (e.g., fatty acid metabolism), energy and carbohydrate metabolism (e.g., glycolysis; pentose phosphate pathway; starch and sucrose metabolism), secondary metabolism (e.g., flavone and flavonol biosynthesis; biosynthesis of terpenoids and steroids), and transport and stress response (e.g., ABC transporters; plant hormone signal transduction).

**Figure 6:**
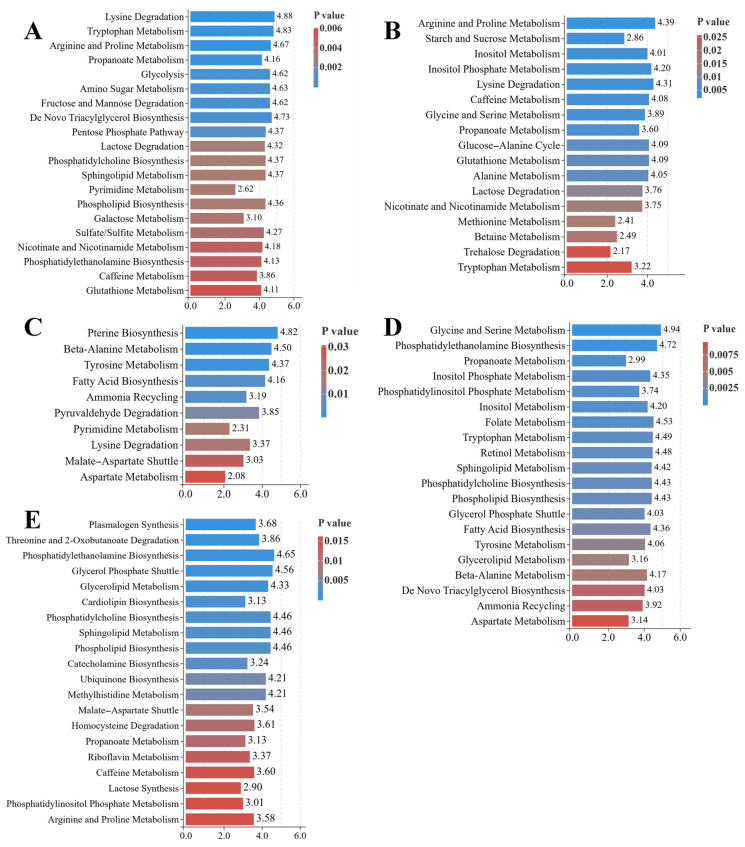
MSEA of differentially accumulated metabolites in different Hainan Dayezhong black teas. (**A**) DY1-vs-DY2, (**B**) DY2-vs-DY3, (**C**) DY1-vs-DY4, (**D**) DY2-vs-DY4, (**E**) DY4-vs-DY5.

To further elucidate the overall patterns of metabolite changes within these pathways, metabolite set enrichment analysis (MSEA) was performed on metabolites [47]. MSEA results confirmed significant perturbations in the aforementioned core metabolic categories, which included energy and carbohydrate metabolism (e.g., the glucose–alanine cycle), amino acid and derivative metabolism, lipid metabolism (e.g., phosphatidylcholine biosynthesis; phosphatidylethanolamine biosynthesis; phospholipid biosynthesis; phosphatidylinositol phosphate metabolism), nucleotide and cofactor metabolism, and secondary metabolism and stress response (Figure 6). Collectively, these analyses demonstrate that the differences in geographical origin (BS, WZS, QZ) and harvest season (spring, summer) do not merely cause isolated changes in a few metabolites. Instead, they are associated with broad divergence in overall metabolic profiles, potentially through modulating fundamental energy metabolic flux, altering the allocation of carbon and nitrogen resources, and influencing the biosynthetic pathways of key secondary metabolites in tea plants [18,48].

### 3.7. Metabolite Analysis of Dayezhong Black Tea

**Figure 7:**
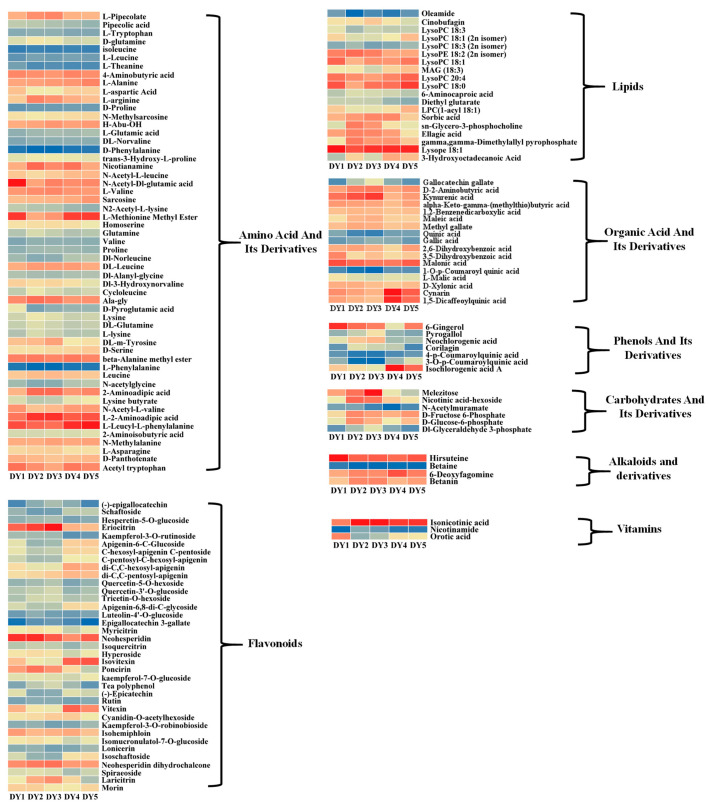
Heatmap of 177 differential metabolites across different Hainan Daye black tea samples (DY1, DY2, DY3, DY4, DY5). The color intensity indicates the relative level of each metabolite, with color gradients reflecting differences in metabolite abundance among the groups.

Environmental and seasonal factors are associated with significant variations in non-volatile metabolites of tea leaves. As shown in Figure 7, a total of 177 differential metabolites were screened across all samples, primarily including amino acids and derivatives (62 types), flavonoids (37 types), lipids (18 types), organic acids and derivatives (17 types), phenols and derivatives (8 types), carbohydrates and derivatives (6 types), alkaloids and derivatives (4 types), and vitamins (3 types). Amino acids and their derivatives represented the most abundant category, indicating their prominent contribution to the metabolic profile and flavor characteristics of HDZBT.

Amino acids are key taste-active compounds determining the flavor profile of tea infusion. A variety of amino acids were identified in the samples, including umami amino acids (e.g., L-aspartic acid, L-glutamic acid, L-theanine, glutamine, asparagine), sweet amino acids (e.g., L-alanine, D-proline, L-proline, L-serine), and bitter amino acids (e.g., L-arginine, L-isoleucine, L-leucine, L-tryptophan, L-phenylalanine). Among them, L-theanine, L-glutamic acid and L-aspartic acid constitute the core substances responsible for the refreshing and mellow taste of tea infusion; their accumulation may be linked to the glutamine synthetase–glutamate synthase (GS-GOGAT) cycle [38]. Quantitative analysis revealed indicative accumulation patterns: L-pipecolic acid, DL-norleucine and cycloleucine were extremely highly accumulated in sample DY1, and could serve as potential geographical markers. Furthermore, the influences of harvest season and plucking position were context-dependent on geographical origin: within the WZS region, the total amino acid content and taste coordination ratio of the spring sample (DY3) were slightly higher than those of the summer sample (DY2). This is consistent with previous findings on early and late spring black teas [11]. Among summer samples from QZ, the bud-tip sample (DY5) exhibited higher L-theanine content and taste coordination ratio than the one-bud-and-two-leaves sample (DY4).

Flavonoids represent another important category of differential metabolites [35]. Ester-type catechins, such as EGC and EGCG, are the primary contributors to the astringency and antioxidant activity of tea infusion, and their content trend in DY1 aligns with the results shown in Figure 3. Notably, flavonoid C-glycosides, including schaftoside and isovitexin, exhibited specific high accumulation in WZS samples, and may represent characteristic components of this origin. In contrast, flavonoid O-glycosides, such as luteolin-4′-O-glucoside and isoquercitrin, were generally present at higher levels in DY4, which may be related to the seasonal metabolism or processing transformation of this sample and contributes to a stronger bitter undertone.

Although lipids occur at relatively low content in tea leaves, they are crucial substances influencing tea quality formation, processing transformation and aroma generation [4]. The lipids identified in this study include oleamide, lysophosphatidylcholine (LysoPC), lysophosphatidylethanolamine (LysoPE), monoacylglycerol (MAG), and free fatty acids such as sorbic acid. Among these, phosphatidylcholine and phosphatidylethanolamine, as the major structural phospholipids of cell membranes, can undergo enzymatic or thermal degradation during processing to generate lysophospholipids and free fatty acids, thereby affecting tea flavor and quality [29]. This study found that lysophosphatidylcholine levels were generally higher in DYZBT samples from WZS (DY2 and DY3) than those from BS and QZ areas. Notably, the spring sample DY3 demonstrated a specific accumulation advantage in key aroma precursors and functional components. Specifically, the contents of monoacylglycerol (MAG, 18:3) and γ,γ-dimethylallyl pyrophosphate (DMAPP) in DY3 were the highest among all samples. MAG serves as an indirect precursor for fatty acid-derived aromas, such as green and floral notes [49], while DMAPP is the precursor unit for terpenoid aroma biosynthesis (e.g., linalool, nerolidol) [50]. The concurrent high-level accumulation of these two compounds indicates that DY3 possesses a richer substrate foundation for aroma synthesis prior to processing, providing material assurance for the formation of its elegant and complex aroma profile. Furthermore, ellagic acid content in DY3 was significantly higher than that in the summer sample DY2 from the same origin. As a known potent natural antioxidant, high ellagic acid content may enhance the potential health benefits of this tea, further strengthening its functional advantages as a spring-harvested tea.

Analysis of organic acids and their derivatives revealed a dramatically different distribution of cynarin and 1,5-dicaffeoylquinic acid among samples. Both compounds were present at extremely low levels in samples from the QZ producing area (DY4 and DY5), while maintaining relatively high levels in samples from BS and WZS (DY1-DY3). This distribution pattern indicates their potential as characteristic chemical markers for distinguishing QZ-origin teas from other regions. Among the carbohydrates and derivatives, D-fructose 6-phosphate, D-glucose-6-phosphate and D-glyceraldehyde 3-phosphate function as key nodal molecules in sugar metabolic pathways, associated with glycolysis, the pentose phosphate pathway and the Calvin cycle, respectively [39]. Their collective abundance reflects the intensity of primary metabolic flux and the direction of carbon allocation in tea plants. These intermediates provide precursors for synthesizing secondary metabolites such as amino acids, organic acids and aroma compounds, thus potentially contributing to the formation of final tea quality and aroma characteristics [34].

## 4. Conclusions

This study systematically clarified the multi-level effects of harvest season, geographical origin and plucking position on the chemical quality of HDZBT. Geographical origin was the primary determinant of tenderness and catechin accumulation, harvest season strongly influenced taste-active and antioxidant components, and plucking position finely regulated specific catechin monomers. Nerolidol, linalool and benzaldehyde were identified as key aromas. Differential metabolites including flavonoid C-glycosides, amino acids, lipids and organic acids can be used as potential characteristic markers to distinguish producing regions and harvesting seasons.

Nevertheless, this study has two main limitations. First, the three pre-harvest factors (geographical origin, harvest season, and plucking position) were not independently controlled, and continuous on-site monitoring of key environmental parameters (temperature, precipitation, sunlight intensity, and soil properties) was not conducted during tea growth. The conclusions only reflect the combined effects of multiple factors, which require further quantification through a full factorial experimental design. Second, this study was limited to descriptive metabolomic analysis without functional validation, and future research needs to integrate multi-omics approaches to elucidate the underlying molecular mechanisms. Despite these limitations, this study systematically characterized the volatile and non-volatile metabolic profiles of HDZBT under the influences of geographical origin, harvest season, and plucking position, and screened key metabolites associated with its sensory quality. This study provides reliable metabolic data and practical reference for optimal harvesting period selection, high-quality raw material screening and quality improvement of local characteristic black tea.

## Figures and Tables

**Table 1:** Description of Hainan Dayezhong black tea samples.

Number	Picking Location	Picking Season	Picking Time	Picking Part
Hainan Dayezhong Tea Sample No. 1 (DY1)	Baisha (BS)	Summer	8 April 2024	One bud and two leaves
Hainan Dayezhong Tea Sample No. 2 (DY2)	Wuzhishan (WZS)	Summer	28 June 2024	One bud and two leaves
Hainan Dayezhong Tea Sample No. 3 (DY3)	Wuzhishan	Spring	5 March 2024	One bud and two leaves
Hainan Dayezhong Tea Sample No. 4 (DY4)	Qiongzhong (QZ)	Summer	10 June 2024	One bud and two leaves
Hainan Dayezhong Tea Sample No. 5 (DY5)	Qiongzhong	Summer	28 April 2024	Bud tip

## Data Availability

The original contributions presented in the study are included in the article and Supplementary Materials; further inquiries can be directed to the corresponding author.

## Supplementary Materials

The following supporting information can be downloaded at https://www.mdpi.com/article/10.3390/foods15122164/s1, Section S1: Non-targeted metabolomics; Figure S1: Composition of volatile components in Hainan Dayezhong black tea; Figure S2: KEGG pathway analysis of differentially accumulated metabolites in different large-leaf black tea samples: (A) DY1-vs-DY2, (B) DY2-vs-DY3, (C) DY1-vs-DY4, (D) DY2-vs-DY4, (E) DY4-vs-DY5; Table S1: The VOCs with ROAV > 1.0 in Hainan Dayezhong black tea; Table S2: Differential metabolites in Hainan Dayezhong black tea samples.
