# Supplementary material for: Pre-Harvest Factors Drive Metabolic and Flavor Variations in Hainan Dayezhong Black Tea
Source: Foods. 2026 Jun 16;15(12):2164. doi: 10.3390/foods15122164 (PMC13298297; doi:10.3390/foods15122164)
Supplement: Supplementary file 1 [file foods-15-02164-s001.zip › foods-4319581-supplementary.pdf]

Pre-Harvest Factors Drive Metabolic and Flavor Variations in Hainan Dayezhong Black Tea

Zongzhuang Fang [1], Xiaoyan Zheng [2], Zhenduan Wang [3], Kai Guo [3], Xingsheng Yue [3] and Shanying Zhang [3,*]

[1] Sanya Research Institute (Hainan Laboratory Animal Research Center), Hainan Academy of Agricultural Sciences, Sanya 572019, China
[2] Institute of Tropical Bioscience and Biotechnology, Chinese Academy of Tropical Agricultural Sciences, Haikou 571101, China
[3] School of Tropical Agriculture and Forestry (School of Agricultural and Rural Affairs, School of Rural Revitalization), Hainan University, Haikou 570228, China
* Correspondence: zhangsy@hainanu.edu.cn

S1. Non-targeted metabolomics

Metabolites Extraction

Tissues (100 mg) were individually grounded with liquid nitrogen and the homogenate was resuspended with pre chilled 80% methanol and 0.1% formic acid by well vortex. The samples were incubated on ice for 5 min and then were centrifuged at 15,000 g, 4℃ for 20 min. Some of supernatant was diluted to final concentration containing 53% methanol by LC-MS grade water. The samples were subsequently transferred to a fresh Eppendorf tube and then were centrifuged at 15000 g, 4℃ for 20 min. Finally, the supernatant was injected into the LC-MS/MS system analysis.

HPLC-MS/MS Analysis

LC-MS/MS analyses were performed using an ExionLC™ AD system (SCIEX) coupled with a QTRAP® 6500+ mass spectrometer (SCIEX) in Genedenovo (Guangzhou, China). Samples were injected onto a Xselect HSS T3 (2.1×150 mm, 2.5 μm) using a 20-min linear gradient at a flow rate of 0.4 mL/min for the positive/negative polarity mode. The eluents were eluent A (0.1% Formic acid-water) and eluent B (0.1% Formic acid-acetonitrile). The solvent gradient was set as follows: 2% B, 2 min; 2-100% B, 15.0 min; 100% B, 17.0 min； 100-2% B, 17.1 min； 2% B, 20min. QTRAP® 6500+ mass spectrometer was operated in positive polarity mode with Curtain Gas of 35 psi, Collision Gas of Medium, Ion Spray Voltage of 5500V, Temperature of 550℃, Ion Source Gas of 1：60, Ion Source Gas of 2：60. QTRAP® 6500+ mass spectrometer was operated in negative polarity mode with Curtain Gas of 35 psi, Collision Gas of Medium, Ion Spray Voltage of -4500V, Temperature of 550℃, Ion Source Gas of 1：60, Ion Source Gas of 2：60.

Metabolites Identification and Quantification

The detection of the experimental samples using MRM (Multiple Reaction Monitoring) were based on house database. The Q3 were used to the metabolite quantification. The Q1, Q3, RT (retention time), DP (declustering potential) and CE (collision energy) were used to the metabolite identification. The data files generated by HPLC-MS/MS were processed using the SCIEX OS Version 1.4 to integrate and correct the peak. The main parameters were set as follows: minimum peak height, 500; signal/noise ratio, 5; gaussian smooth width, 1. The area of each peak represents the relative content of the corresponding substance.

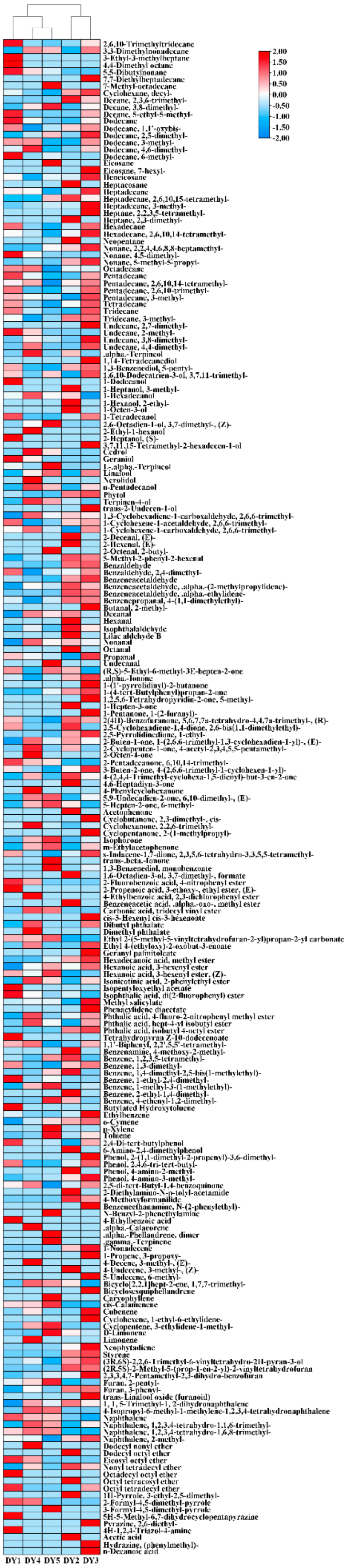

Figure S1 Composition of volatile components in Hainan Dayezhong black tea

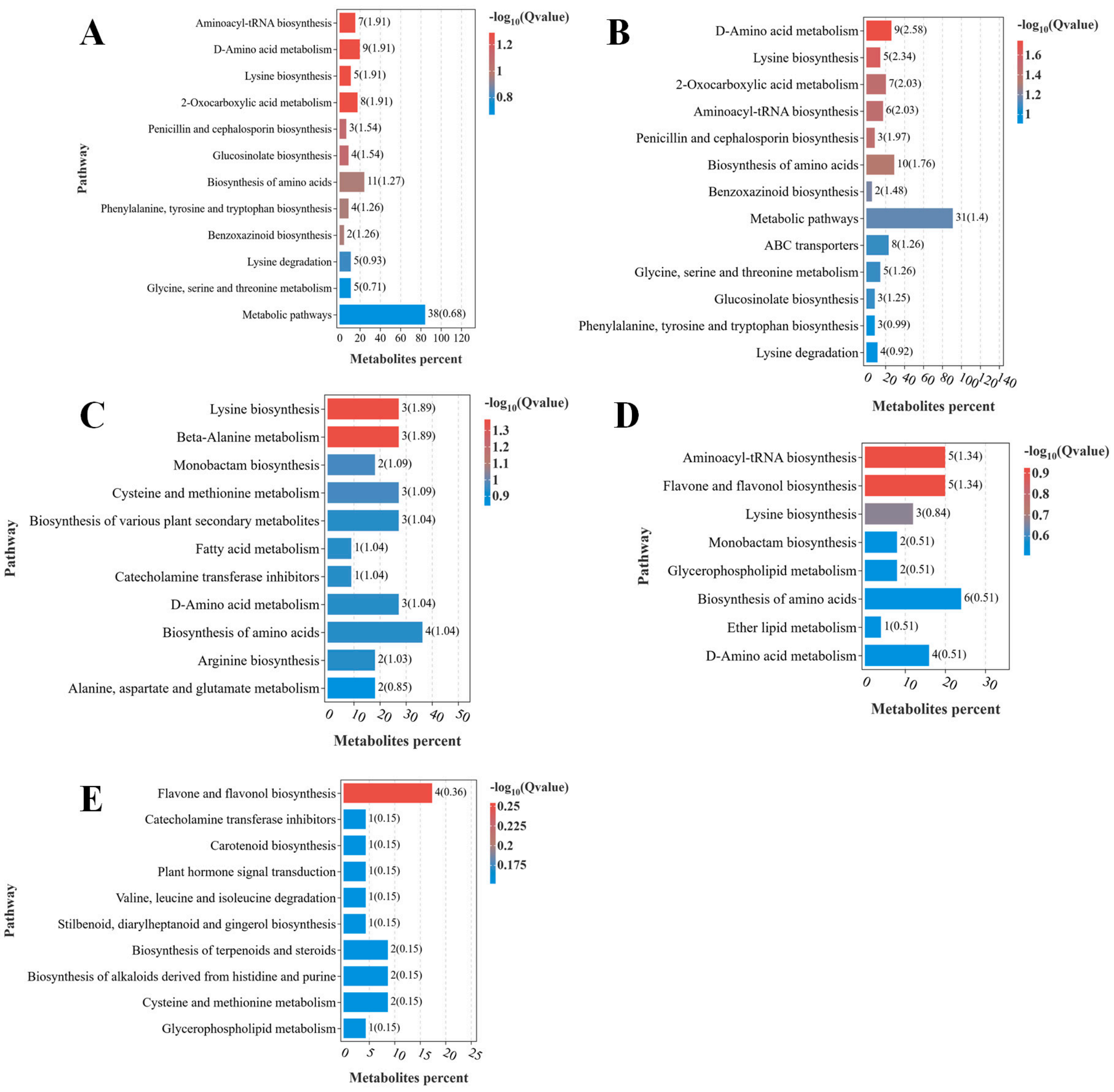

Figure S2 KEGG pathway analysis of differentially accumulated metabolites in different large-leaf black tea samples: (A) DY1-vs-DY2, (B) DY2-vs-DY3, (C) DY1-vs-DY4, (D) DY2-vs-DY4, (E) DY4-vs-DY5

Table S1 The VOC with ROAV > 1.0 in Hainan Dayezhong black tea

No	Compounds	Class	CAS	Threshold (μg/L)	Odor description[1]	ROAV DY1	DY2	DY3	DY4	DY5
1	Pentadecane	Alkane	4643-27-0	200	waxy, alkane-like	0.3667	0.2690	0.0848	0.1038	0.0595
2	Tetradecane	Alkane	629-59-4	16	waxy, alkane-like	5.7103	5.2871	1.5243	1.6614	0.9939
3	α-Terpineol	Alcohol	98-55-5	330	floral, woody, citrusy,	0.0000	0.0339	0.0000	0.0230	0.0000
4	1,3-Benzenediol, 5-pentyl-	Alcohol	3391-86-4	26	earthy	2.5092	2.5320	0.7029	0.5231	0.3180
5	1,6,10-Dodecatrien-3-ol, 3,7,11-trimethyl- (nerolidol)	Alcohol	7212-44-4	0.02	floral, woody, green	1247.8355	756.5645	384.7507	316.4074	289.7773
6	Lauryl alcohol	Alcohol	104-76-7	270	fatty, floral, soapy	0.0118	0.0000	0.0000	0.0000	0.0000
7	1-Octen-3-ol	Alcohol	3391-86-4	1	earthy	0.0000	4.9365	0.0000	0.0000	0.0000
8	Myristyl alcohol	Alcohol	98-86-2	10	faint waxy, soapy, or fatty	1.4532	0.8311	0.3341	0.2097	0.1357
9	cis-Nerolidol	Alcohol	87-44-5	64	floral, woody, green	0.0000	0.0000	0.0000	0.1767	0.4382
10	2-Ethyl-1-hexanol	Alcohol	104-76-7	4.5	sweet, floral, oily	0.0000	0.0000	0.0000	0.4969	0.0000
11	Geraniol	Alcohol	106-24-1	7.5	Sweet, floral (rose-like), fruity	10.8950	0.0000	0.0000	1.3005	0.0000
13	Linalool	Alcohol	78-59-1	6	floral, sweet, woody	100.0000	100.0000	100.0000	100.0000	100.0000
14	Phytol	Alcohol	150-86-7	590	balsamic, green	0.0056	0.0218	0.0064	0.0000	0.0000
15	2-Decenal, (E)-	Aldehyde	3913-81-3	17	orange peel, fatty	0.0000	1.8176	0.0000	0.0000	0.0000
16	2-Hexenal, (E)-	Aldehyde	6728-26-3	40	green, leafy, apple-like,	0.0000	0.1937	0.0000	0.0000	0.0000
17	2-Octenal, 2-butyl-	Aldehyde	98-55-5	86	fatty, waxy	0.0000	0.0000	0.0000	0.0000	0.0143
18	Benzaldehyde	Aldehyde	527-84-4	5	almond	26.1312	62.0637	16.0266	7.2422	4.8818

19	Benzeneacetaldehyde	Aldehyde	36653-82-4	4	honey, floral, green	90.2101	168.4573	54.6646	22.7320	11.9302
20	Decanal	Aldehyde	112-31-2	0.1	waxy, citrusy	0.0000	126.4738	15.1619	43.1092	21.3338
21	Hexanal	Aldehyde	66-25-1	300	green, grassy, leafy	0.0000	0.0274	0.0000	0.0000	0.0000
22	Nonanal	Aldehyde	34995-77-2	190	waxy, citrusy	0.0905	0.3489	0.0703	0.1443	0.0697
23	Octanal	Aldehyde	124-13-0	0.7	waxy, citrus (lemon), fatty	0.0000	6.7306	0.0000	0.0000	0.0000
24	Propanal	Aldehyde	100-42-5	730	fruity, aldehydic	0.0416	0.0192	0.0137	0.0125	0.0075
25	.alpha.-Ionone	Ketone	127-41-3	270	woody, floral	0.0529	0.0647	0.0192	0.0130	0.0118
26	1-Hepten-3-one	Ketone	108-88-3	140	earthy, green	0.0000	0.0262	0.0000	0.0000	0.0000
27	β-Damascenone	Ketone	100-41-4	26	sweet, fruity (apple, plum), floral	0.8130	1.9015	0.3935	0.7085	0.3434
28	2-Octen-4-one	Ketone	4643-27-0	200	earthy, musty	0.0000	0.0000	0.0000	0.0044	0.0000
29	β-ionone	Ketone	14901-07-6	5.7	floral, woody, sweet, fruity	23.1786	22.6272	8.1787	6.0286	2.9317
31	Acetophenone	Ketone	98-86-2	10	sweet, floral (orange blossom), almond	0.0000	0.7812	0.0000	0.0000	0.0000
32	Dibutyl phthalate	Ester	84-74-2	70	faint, oily	0.0000	0.1300	0.1633	0.2481	0.0385
33	Methyl salicylate	Ester	119-36-8	10	wintergreen, minty, sweet	69.3219	42.7943	32.9374	26.0563	17.4723
34	Dodecane	Alkane	112-40-3	10000	waxy, alkane-like	0.0041	0.0023	0.0008	0.0010	0.0006
35	Ethylbenzene	Aromatic Hydrocarbon	106-25-2	290	sweet, aromatic	0.0410	0.0371	0.0332	0.0065	0.0038
36	o-Cymene	Terpenoids	527-84-4	10	spicy, herbaceous	0.3233	0.9117	0.2562	0.2276	0.1905
37	Toluene	Aromatic Hydrocarbon	108-88-3	300	sweet, pungent	0.0000	0.0234	0.0150	0.0034	0.0318

38	.alpha.-Phellandrene, dimer	Terpenoids	99-83-2	1	woody, balsamic, pine-like	0.0000	0.0000	0.0000	0.0000	0.1211
39	.gamma.-Terpinene	Terpenoids	99-85-4	50	citrus, lemon, pine, herbaceous	0.0000	0.0000	0.0000	0.0000	0.0341
40	Caryophyllene	Terpenoids	87-44-5	500	woody, spicy, clove-like	0.0000	0.0000	0.0000	0.0000	0.0037
41	D-Limonene	Terpenoids	5989-27-5	34	citrus (orange), lemon-like, fresh	0.6559	1.0883	0.2082	0.1992	0.4308
43	Limonene	Terpenoids	14901-07-6	8.4	citrus (orange, lemon), fresh	0.0000	0.0000	0.0000	0.6814	0.0000
44	Styrene	Aromatic Hydrocarbon	100-42-5	3	aweet, floral	0.0000	9.2300	2.9477	0.0000	0.0000
45	Furan, 2-pentyl-	Heterocycle	3777-69-3	4	green bean, fruity	2.7385	0.0000	0.1878	1.4291	1.8903
46	trans-Linalool oxide	Ether	119-36-8	320	floral, woody, sweet	0.0000	0.0000	0.0428	0.0000	0.0000

[1] The Odor descriptions are from http://www.thegoodscentscompany.com, http://perflavory.com/, http://www.odour.org.uk/odour/index.html, http://foodflavorlab.cn/#/home
